# Supplementary material for: Seminoma and Embryonal Carcinoma Footprints Identified by Analysis of Integrated Genome-Wide Epigenetic and Expression Profiles of Germ Cell Cancer Cell Lines
Source: PLoS One. 2014 Jun 2;9(6):e98330. doi: 10.1371/journal.pone.0098330 (PMC4041891; doi:10.1371/journal.pone.0098330)
Supplement: File S1 — ZIP file containing DMRforPairs output for significant regions. Please start from the html files. (ZIP) [file pone.0098330.s008.zip › figures/13789.pdf]

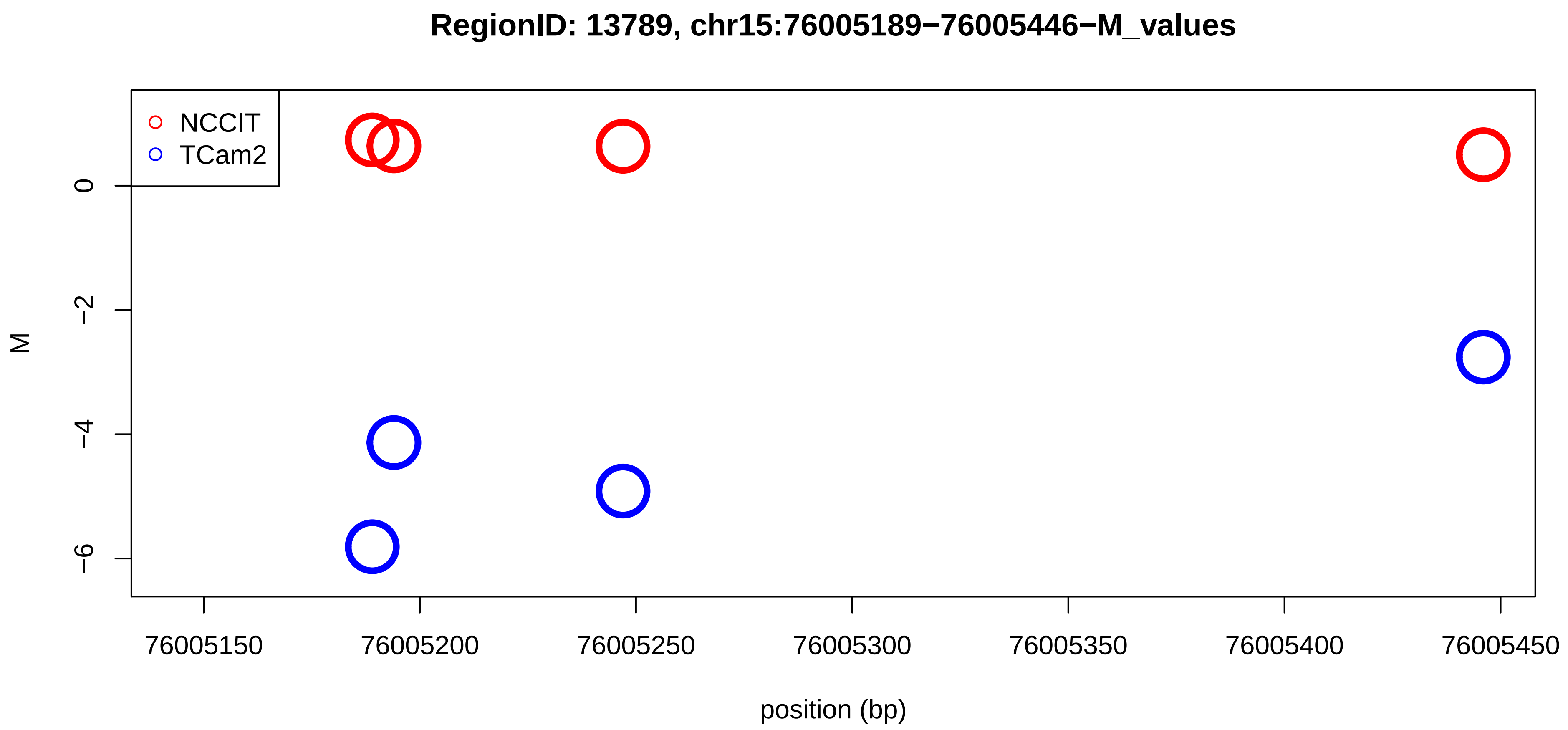
RegionID: 13789, chr15:76005189−76005446−M_values
NCCIT
TCam2
M
0
−2
−4
−6
76005150
76005200
76005250
76005300
76005350
76005400
76005450
position (bp)

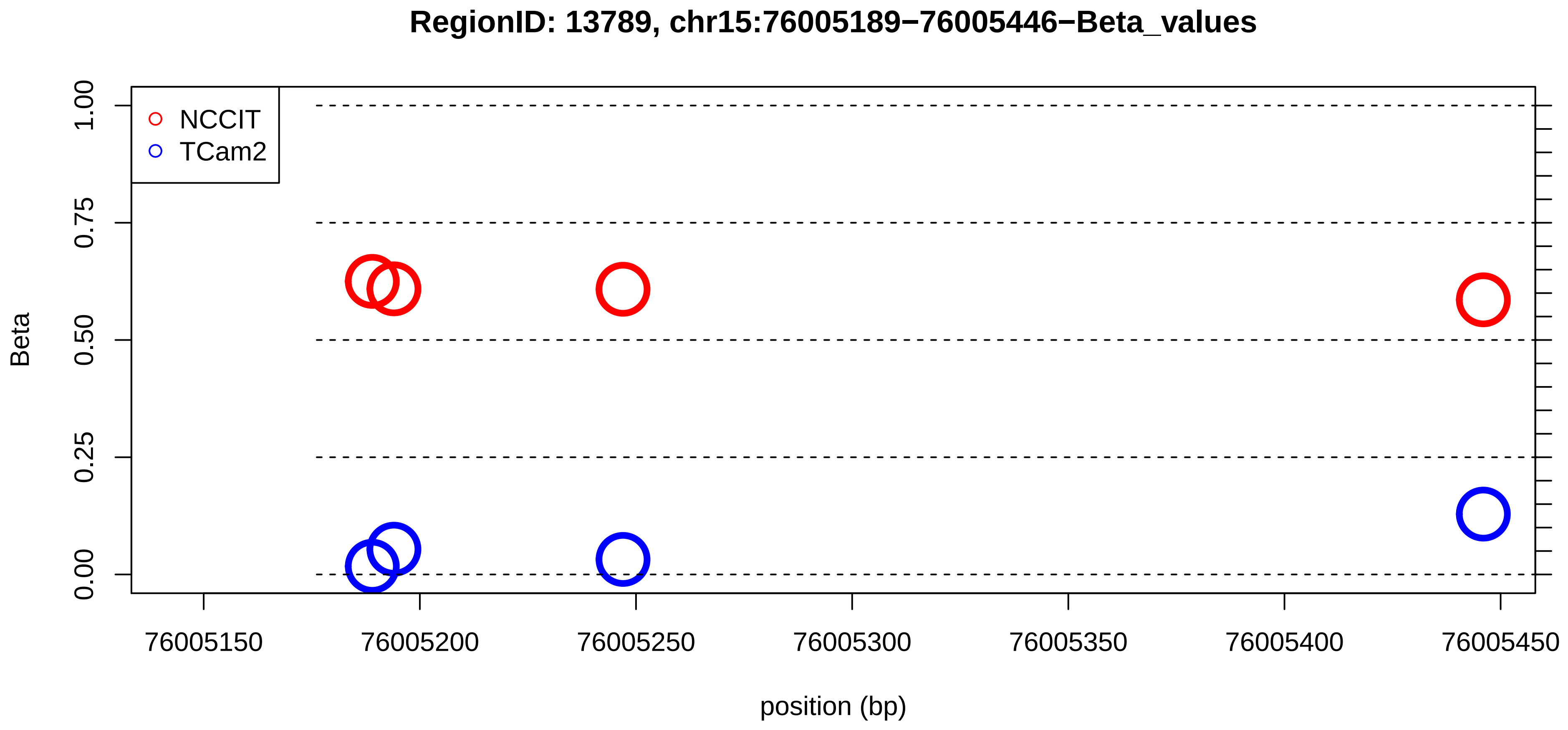
RegionID: 13789, chr15:76005189−76005446−Beta_values
NCCIT
TCam2
Beta
1.00
0.75
0.50
0.25
0.00
76005150
76005200
76005250
76005300
76005350
76005400
76005450
position (bp)
